# Evidence of Coexistence of C_3_ and C_4_ Photosynthetic Pathways in a Green-Tide-Forming Alga, *Ulva prolifera*

**DOI:** 10.1371/journal.pone.0037438

**Published:** 2012-05-16

**Authors:** Jianfang Xu, Xiao Fan, Xiaowen Zhang, Dong Xu, Shanli Mou, Shaona Cao, Zhou Zheng, Jinlai Miao, Naihao Ye

**Affiliations:** 1 Key Laboratory of Marine Bioactive substance, The First Institute of Oceanography, State Oceanic Administration, Qingdao, China; 2 Yellow Sea Fisheries Research Institute, Chinese Academy of Fishery Sciences, Qingdao, China; 3 Qingdao Agricultural University, Qingdao, China; Laurentian University, Canada

## Abstract

*Ulva prolifera*, a typical green-tide-forming alga, can accumulate a large biomass in a relatively short time period, suggesting that photosynthesis in this organism, particularly its carbon fixation pathway, must be very efficient. Green algae are known to generally perform C_3_ photosynthesis, but recent metabolic labeling and genome sequencing data suggest that they may also perform C_4_ photosynthesis, so C_4_ photosynthesis might be more wide-spread than previously anticipated. Both C_3_ and C_4_ photosynthesis genes were found in *U. prolifera* by transcriptome sequencing. We also discovered the key enzymes of C_4_ metabolism based on functional analysis, such as pyruvate orthophosphate dikinase (PPDK), phosphoenolpyruvate carboxylase (PEPC), and phosphoenolpyruvate carboxykinase (PCK). To investigate whether the alga operates a C_4_-like pathway, the expression of *rbc*L and PPDK and their enzyme activities were measured under various forms and intensities of stress (differing levels of salinity, light intensity, and temperature). The expression of *rbc*L and PPDK and their enzyme activities were higher under adverse circumstances. However, under conditions of desiccation, the expression of *rbc*L and ribulose-1, 5-biphosphate carboxylase (RuBPCase) activity was lower, whereas that of PPDK was higher. These results suggest that elevated PPDK activity may alter carbon metabolism and lead to a partial operation of C_4_-type carbon metabolism in *U. prolifera*, probably contributing to its wide distribution and massive, repeated blooms in the Yellow Sea.

## Introduction

Carbon fixation is an important biological process in all photosynthetic organisms. C_4_ plants are characterized by high rates of photosynthesis and efficient use of water and nitrogen resources [1]. High photosynthetic rates are achieved by addition of a new metabolic pathway, the C_4_ cycle, in which the initial product of CO_2_ fixation is a four-carbon (C) organic acid rather than a three-carbon (C) acid. C_4_ plants show drastically reduced rates of photorespiration because CO_2_ is concentrated at the site of Rubisco and is able to outcompete molecular oxygen, which, when used by Rubisco, results in photorespiration [2]. The C_4_ photosynthetic carbon cycle is an elaborated addition to the C_3_ photosynthetic pathway, which ensures high rates of photosynthesis even when CO_2_ concentrations are low. C_4_ photosynthesis evolved several times independently during the evolution of higher plants. It originated at least 32 times in eudicots and 16 times in monocots [3]. It had evolved from ancestral C_3_ plants via a series of anatomical and physiological adaptations to high light intensities, high temperatures, low *p*CO_2_, and dryness [4].

In aquatic environments, [CO_2_] can be a primary limitation for photosynthesis because of the low capacity of water to hold gaseous CO_2_ and the slow diffusion rate of dissolved molecules [5], [6]. It has been demonstrated that many aquatic photosynthetic organisms can take up both CO_2_ and HCO_3_^−^ from the surrounding media, and this capacity is greatly strengthened under CO_2_-limiting conditions, including the atmospheric pressure of CO_2_. This system is generally known as the inorganic carbon-concentrating mechanism (CCM) [7]. Cyanobacteria, algae, and some angiosperms evolved multiple mechanisms to actively accumulate inorganic carbon around Rubisco by use of membrane transporters and carbonic anhydrases [8]. The aquatic environment is home to as great a diversity of photosynthetic pathways as terrestrial environments, and there exist C_3_, C_4_, CAM, and C_3_–C_4_ photosynthetic pathways [9]. Although apparently lacking Kranz anatomy, aquatic *Orcuttia californica* (an aquatic embryophyte) could also conduct C_4_ photosynthesis [9]. Some species, such as *Chara contraria* (a charophyte green algae), *Marsilea vestita* (an embryophyte), *Eleocharis acicularis* (an embryophyte) and *Pilularia Americana* (an embryophyte), have both C_3_ and C_4_ fixation in aquatic habitats [9]. Alterations of photosynthetic pathways under environmental stress have been suggested to contribute to the adaptation of plants to environmental stress [10]. For example, *Hydrilla verticillata*, a submerged aquatic plant, changes its photosynthetic pathway from C_3_ to C_4_ under conditions of CO_2_ deficiency [11]. Therefore, environmental factors are of critical importance in the change of photosynthetic pathways.

From many studies on primary photosynthetic carbon metabolism, it is believed that the operation of the Calvin–Benson cycle (C_3_ cycle) is predominant in algae [12], [13]. However, recent papers have reported evidence for the operation of C_4_ photosynthesis as an alternative CCM in the marine diatom *Thalassiosira weissflogii* [14]–[17]. The case for C_4_ photosynthesis has been further strengthened by the occurrence of relevant genes in recently sequenced marine phytoplankton genomes, including the diatoms *Thalassiosira pseudonana* and *Phaeodactylum tricornutum* and the green alga *Ostreococcus tauri* and *Micromonas* [18]–[22]. *Ostreococcus* has all the machinery necessary to perform C_4_ photosynthesis. This includes a plastid-targeted NADP(1)-dependent malic enzyme and a phosphoenolpyruvate carboxylase [22]. However, conflicting experimental data shedding doubt on C_4_ photosynthesis in diatoms have been reported [16], [17], and genomic data do not fully clarify the presence and localization of the enzymes that may drive this mechanism [23], [24]. No clear evidence for such C_4_-like processes have been found in the marine diatoms *P. tricornutum* and *T. pseudonana*, for which whole genome sequences are available [25]. The general occurrence of C_4_-like mechanisms in diatoms is therefore still in question [7], [16].

As a special type of harmful algal blooms (HABs), green tides have been increasing in severity and geographic range and are now of growing concern globally. Green tides are vast accumulations of unattached green macroalgae usually associated with eutrophied marine environments [26], [27]. The great majority of green tides are reported to consist of members of just one genus, *Ulva* (some of the species formerly known as *Enteromorpha*) [28], [29]. *Ulva prolifera*, a representative green-tide-forming macroalga [26], is the dominant *Ulva* species along the coastline of the Yellow Sea between June and August [30], [31]. *U. prolifera*, as an intertidal macroalga, can tolerate various kinds of abiotic stresses, including desiccation, changes in temperature and salinity, and exposure to high levels of solar radiation during low tide [32]. Furthermore, the evolutionary status of intertidal pluricellular green algae is between the unicellular green algae and lower land plants, which is an important stage during evolution [33].

It has been proved that marine algae contain C_4_-Pathway, including *Ulva* species [34]. Kremer and Küppers (1977) found that the percentage of malate and aspartate usually accounts for distinctly less than 10% of the total ^14^C-labelling in three *Ulva* species, and these findings were consistent with data from enzymatic analyses, since 86–90% of the carboxylation capacity was due to ribulose-l.5-biphosphate carboxylase in those green algae [34]. Moreover, the occurrence of PEP-C besides RubP-C has been reported from *Ulva* using ^14^C-labelling technique [35], [36]. One of the most standard comparisons of differences in isotopic ratios is the comparison of ^13^C to ^12^C in plants to determine photosynthetic pathway of plants. C_3_ and C_4_ plants have different δ^13^C values, −28.1±2.5‰, −13.5±1.5‰ respectively [37]. Among C_3_ and C_4_ plants, δ^13^C variation can range from 2–5‰. Previous research approved that *Ulva* are C_4_ species since there δ^13^C values are in the range of −14±4‰ [38], [39].

In this study we used next generation sequencing (NGS) technology confirmed the existence of genes necessary for a C_4_ pathway in *U. prolifera*, and we then chose to compare transcript abundance of *U. prolifera* with that of the closest relative, *U. linza*, which has been confirmed to possess the C_4_ pathway (unpublished data). Subsequently, we focused on the expression profile of two key enzymes, namely RuBPCase and PPDK. Ribulose-1, 5-biphosphate carboxylase, a key enzyme of the C_3_ pathway, catalyzes the first major step in carbon fixation. Pyruvate orthophosphate dikinase, a cardinal enzyme of the C_4_ pathway, catalyzes the regeneration of phosphoenolpyruvate (PEP), the primary carboxylation substrate from pyruvate, Pi, and ATP [40]. The rate of PEP formation by PPDK is the lowest in the C_4_ pathway; therefore, this reaction is considered to be the rate-limiting step in the C_4_ pathway [41]. Our results demonstrate that *U. prolifera* may be either a C_3_–C_4_ intermediate species or a C_3_ species displaying C_4_ metabolic characteristics. The involvement of C_4_ metabolism in *U. prolifera* might account for the boom of green tide.

## Materials and Methods

### Sample collection and culture conditions

Floating specimens of *U. prolifera* were collected in the Yellow Sea during the green tide bloom in 2011. In the laboratory, the intact samples were washed several times with sterile seawater, sterilized with 1% sodium hypochlorite for 2 min, and then rinsed with autoclaved seawater. The sterilized material was then placed into an aquarium (d = 40 cm, h = 30 cm) containing enriched and continually aerated seawater (500 µM NaNO_3_ and 50 µM NaH_2_PO_4_) and maintained at 15°C under a 12∶12 h LD photoperiod with 50 µmol photons m^−2^ s^−1^ provided by cool-white fluorescent tubes.

### Stress treatments

*U. prolifera* was exposed to different kinds of stress, namely desiccation and differing levels of salinity, light intensity, and temperature. For desiccation stress, the alga were cultured at 50 µmol photons m^−2^ s^−1^ for different durations (0, 1, 2, 3, 4, and 5 h). Salinity stress consisted of subjecting the organism for 3 h to different salt concentrations (0‰, 15‰, 30‰, 45‰, and 60‰); In light intensity treatment, the samples were exposure to 0, 50, 100, 300, 600, 1000, and 2000 µmol photons m^−2^ s^−1^ for 3 h. For the three forms of stress, temperature was constant at 15°C, and light intensity during the salinity treatment and the temperature treatment was maintained at 50 µmol photons m^−2^ s^−1^. For temperature stress, the materials were cultured at 5, 10, 15, 20, 25, 30 and 35°C for 3 h. Following each stress treatment, *rbc*L and PPDK mRNA expression level was measured using qPCR, RuBPCase and PPDK activity assessed, and Fv/Fm and Y(II) determined using Dual-PAM-100 (Walz GmbH, Germany).

### Light and transmission electron microscopy

The sample preparation was finished according to the methods mentioned by Chen et al. [42] It consisted of the following steps: collecting the algal; fixing with 1% (v/v) glutaraldehyde and postfixing with 1%(v/v) osmium tetroxide both in sterilizing seawater; dehydration in a series of acetone solutions; suspension in the mixture of epoxy resin (Epon812) and acetone; embedded in 100% Epon812; polymerized and sectioned using a LeicaUC6 ultra microtome; picked up on 200-mesh copper grids and post-stained with urinal acetate. Finally, the sections were examined under an optical microscope (Nikon Eclipse 80i) and a transmission electron microscopy (Hitachi H-7650) at an accelerating voltage of 60 kv.

### Transcriptome sequencing

The alga were treated with different stress conditions, such as low temperature (6°C, 2 h), high temperature (42°C, 1 h), high light (1000 µmol photons m^−2^ s^−1^, 1 h), high salt (93‰, 3 h) and UV-B stress (60 µw cm^−2^, 3 h). Total RNA of all treated samples was extracted and purified, followed by synthesis and purification of double-stranded cDNA and sequencing of cDNA using a Roche GS FLX Titanium platform. To reconstruct the metabolic pathways in *U. prolifera*, high-quality reads were assigned to the Kyoto Encyclopedia of Genes and Genomes (KEGG) using the software package MEGAN (version 4.0) [43].

### Sequence Analysis

The partial *rbc*L cDNA sequence acquired from GenBank and the cDNA open reading frame (ORF) sequence of PPDK obtained from transcriptome sequencing, were examined for homology with other known sequences using the BLAST X program available at the website of the National Center for Biotechnology Information <www.ncbi.nlm.nih.gov/blast>. We used the Six Frame Translation of Sequence system <http://searchlauncher.bcm.tmc.edu/seq-util/Options/sixframe.html> analyzing deduced amino acid sequence. Multiple sequence alignments were generated using the program CLUSTAL X and then analyzed using the program BioEdit [44], [45]. A phylogenetic tree was constructed using the neighbor-joining algorithm of the MEGA 4.0 program [46], [47].

### Real-time quantitative PCR

Total RNA of *U. prolifera* exposed to each form and level of stress was extracted using TRIzol reagent (Invitrogen, Carlsbad, CA, USA) as specified in the user manual and dissolved in diethypyrocarbonate (DEPC)-treated water. The cDNA used for real-time quantitative PCR was synthesized from the total RNA using Moloney murine leukemia virus reverse transcriptase (Promega Biotech Co., Madison, Wisconsin, USA).

The real-time quantitative PCR reactions were performed with the ABI StepOne Plus Real-Time PCR System (Applied Biosystems, USA) using SYBR Green fluorescence (TaKaRa) according to the manufacturer's instructions. To normalize the relative expression of the selected genes, an 18S rDNA gene was used as reference. Three pairs of gene-specific primers (Table 1) were designed according to the *rbc*L cDNA, PPDK cDNA, and 18S rDNA sequences using Primer Express 3.0. For each selected gene, three biological replicates were assayed independently. The qPCR amplifications were carried out in a total volume of 20 µL containing 10 µl of 2× SYBR Premix Ex TaqTM II (TaKaRa Biotech Co., Dalian, China), 0.6 µl (10 µM) of each primer, 2.0 µl of the diluted cDNA mix, and 6.8 µl de-ionized water. The qPCR amplification profile was obtained as follows: 95°C for 30 s followed by 40 cycles of 95°C for 5 s, 60°C for 10 s, and 72°C for 40 s. The 2^−ΔΔCT^ method [48] was used to analyze the quantitative real-time PCR data.

### Enzyme assays

The activity of RuBP carboxylase and PPDK in *U. prolifera* exposed to the treatments was measured, RuBP carboxylase activity by the method described by Gerard and Driscoll and PPDK activity by that described by Sayre et al. [49], [50]; both methods were modified as required.

**Table 1:** Primers used in the qPCR assay.

Name		Primers Sequence (5′-3′)
*rbc*L	F	TACAAATCTCAAGCCGAAACTG
	R	AATCTTTAGCAAATTGACCACG
PPDK	F	CACGAACGACCTTACGCAGA
	R	ACGGATCAAACGCCATCAC
18S rDNA	F	ATTAGATACCGTCGTAGTCTCAACC
	R	TCTGTCAATCCTTCCTATGTCTGG

For measuring RuBP carboxylase activity, each sample was ground to a fine powder in liquid nitrogen and homogenized in pre-cooled rubisco extraction solution (1 ml g^−1^ fresh weight), pH 7.6, containing 40 mM Tris-HCl buffer with 10 mM MgCl_2_, 0.25 mM EDTA, and 5 mM reduced glutathione. The homogenate was centrifuged at 10 000 *g* for 10 min at 4°C. The activity was measured in a 4.5 ml cuvette by adding 3 ml of a reaction mixture containing 0.2 ml NADH (5 mM), 0.2 ml ATP (50 mM), 0.1 ml enzyme extract, 0.2 ml creatine phosphate (50 mM), 0.2 ml NaHCO_3_ (0.2 mM), 1.4 ml reaction buffer (0.1 M Tris-HCl buffer, pH 7.8, with 12 mM MgCl_2_ and 0.4 mM EDTA), 0.1 ml creatinephosphokinase (160 units ml^−1^), 0.1 ml phosphoglycerate kinase (160 units ml^−1^), 0.1 ml glyceraldehyde-3-phosphate dehydrogenase (160 units ml^−1^), and 0.3 ml distilled water. The reaction was initiated by adding 0.1 mL ribulose-1, 5-bisphosphate (RuBP) to the reaction cuvette and OD values were recorded every 20 seconds for 3 min by a spectrophotometer at 340 nm. The enzyme activity was expressed in terms of micromoles per gram of fresh weight per minute (µmol g^−1^ FW min^−1^).

For measuring PPDK activity, the samples were ground to a fine powder in liquid nitrogen and homogenized in pre-cooled PPDK extraction solution at pH 8.3 (1 ml g^−1^ fresh weight) containing 100 mM Tris-HCl buffer with 5 mM mercaptoethanol and 2 mM EDTA. The homogenate was centrifuged at 10 000 *g* for 10 min at 4°C. The activity was measured in a 4.5 ml cuvette by adding 3 ml of a reaction mixture containing 0.1 ml Tris-HCl buffer (150 mM, pH 8.3, with 18 mM MgSO_4_), 0.1 ml DTT (300 mM), 0.1 ml PEP (30 mM), 0.1 ml NADH (4.5 mM), 0.1 ml AMP (30 mM), 0.1 ml lactic dehydrogenase (60 units ml^−1^), 0.1 ml enzyme extract, and 1.3 ml distilled water. The reaction was initiated by adding 0.1 mL pyrophosphate natrium to the reaction cuvette and the OD values were recorded every 20 seconds for 3 min at 340 nm. The PPDK activity was also expressed in terms of micromoles per gram of fresh weight per minute (µmol g^−1^ FW min^−1^).

### Chlorophyll uorescence measurements

Photosynthetic performance of *U. prolifera* subjected to the different treatments was measured using Dual-PAM-100. The maximal photochemical efficiency of PS II (Fv/Fm) and the effective PS II quantum yield (Y II) were measured by the method of Fleming et al. [51]. Before measurement, samples were dark adapted for 20 min. Optimal chlorophyll fluorescence quantum yield was calculated according to the following equation: Fv/Fm = (Fm−F_0_)/Fm. Fo and Fm refer to the minimal fluorescence and the maximal fluorescence from dark adapted samples, respectively. Fv is the difference between Fm and Fo. The culture experiments were repeated four times.

## Results

### Transcriptome sequencing

**Figure 1:**
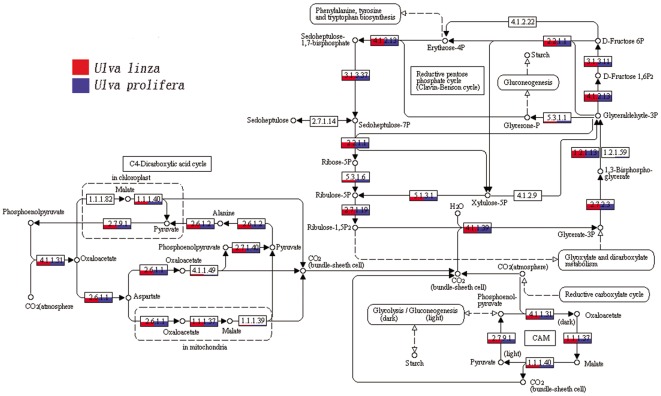
Carbon fixation pathway in *U. linza* and *U. prolifera* generated by KEGG. The numbers within the small boxes are enzyme codes.

We analyzed the carbon fixation pathway in detail and discovered some key genes of enzymes involved in the carbon fixation pathway in *U. prolifera*, such as phosphoenolpyruvate carboxylase, aspartate aminotransferase, ribulose bisphosphate carboxylase, phosphoglycerate kinase, phosphoribulokinase, phosphoenolpyruvate carboxykinase, alanine transaminase, malate dehydrogenase (NADP+), pyruvate orthophosphate dikinase, and pyruvate kinase (Fig. 1), which provided unequivocal molecular evidence that most of the C_3_ pathway, C_4_ pathway, and CAM pathway genes were actively transcribed in *U. prolifera*. Figure 1 shows that both *U. linza* (unpublished) and *U. prolifera* have most of the genes that are indispensable to C_3_ and C_4_ pathways, and the relative enzymes are all the same in both algae. However, the abundances of C_3_ and C_4_ pathway genes in *U. linza* and *U. prolifera* are different. The results suggest the possibility of the existence of two photosynthetic pathways in *U. prolifera*, the Calvin cycle (C_3_) and the Hatch-Slack (C_4_) carbon fixation pathway.

### cDNA Sequence Analysis

**Figure 2:**
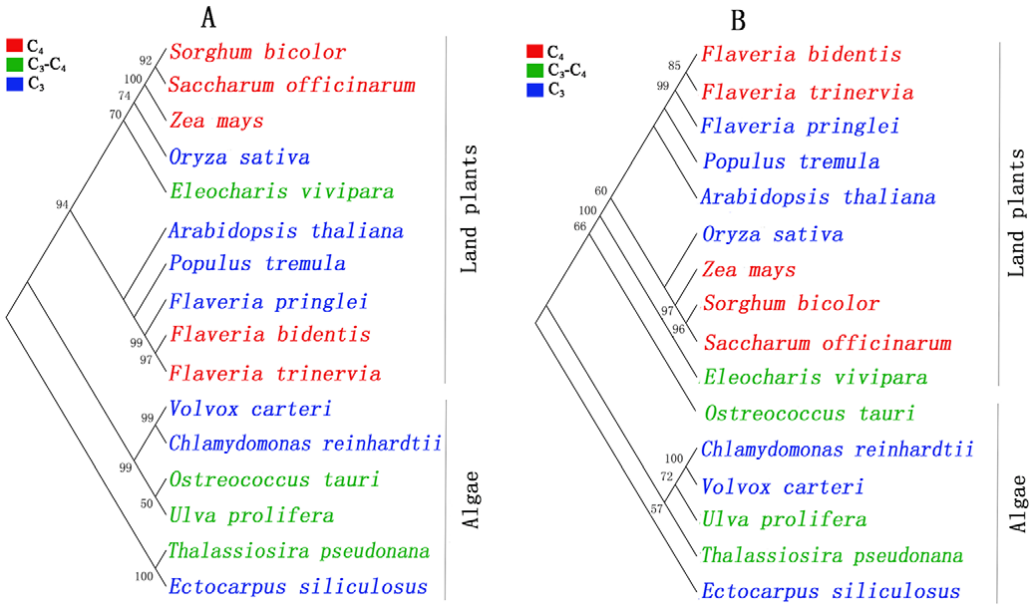
Phylogenetic analysis of *rbc*L and PPDK. The phylogenetic tree was constructed by the neighbor-joining (NJ) method using Mega (version 4.0). Bootstrap analysis was computed with 1000 replicates and bootstrap values below 50% were omitted. C_3_–C_4_ refers to species that possessed the genes for both C_3_ and C_4_ photosynthesis with C_3_ photosynthesis being the primary pathway. (A) Phylogenetic analysis of *rbc*L. GenBank accession numbers of the sequences used for constructing the phylogenetic tree of *rbc*L were as follows: *Ulva prolifera* (FJ042888), *Thalassiosira pseudonana* (YP_874498), *Flaveria bidentis* (ADW80649), *Flaveria trinervia* (ADW80661), *Flaveria pringlei* (ADW80648), *Zea mays* (NP_043033), *Sorghum bicolor* (ABK79504), *Oryza sativa* (CAG34174), *Saccharum officinarum* (YP_054639), *Arabidopsis thaliana* (AAB68400), *Volvox carteri* (ACY06055), *Chlamydomonas reinhardtii* (ACJ50136), *Ostreococcus tauri* (YP_717262), *Ectocarpus siliculosus* (CBH31935), *Populus tremula* (CAD12560), and *Eleocharis vivipara* (CAQ53780). (B) Phylogenetic analysis of PPDK. GenBank accession numbers of the sequences used for constructing the phylogenetic tree of PPDK were as follows: *Ulva prolifera* (JN936854), *Thalassiosira pseudonana* (XP_002290738), *Flaveria bidentis* (AAA86941), *Flaveria trinervia* (CAA55703), *Flaveria pringlei* (CAA53223), *Zea mays* (ADC32810), *Sorghum bicolor* (AAP23874), *Oryza sativa* (CAA06247), *Saccharum officinarum* (AAF06668), *Arabidopsis thaliana* (AEE83621), *Volvox carteri* (XP_002955807), *Chlamydomonas reinhardtii* (XP_001702572), *Ostreococcus tauri* (XP_003075283), *Ectocarpus siliculosus* (CBN74442), *Populus tremula* (CAX83740), and *Eleocharis vivipara* (BAA21654).

The partial *rbc*L cDNA sequence (FJ042888) was acquired from GenBank with a 1305 bp sequence encoding 435 amino acid residues. The PPDK cDNA sequence (JN936854) of ORF was obtained from the *U. prolifera* transcriptome database with a 2700 bp sequence encoding 889 amino acid residues. Phylogenetic analysis was conducted using the amino acid sequences of *rbc*L and PPDK (Fig. 2). The phylogenetic tree of *rbc*L indicated a species clustering that was basically consistent with the evolution of the species, and that of PPDK revealed that the C_4_ pathway had multiple independent origins. In the phylogenetic tree of *rbc*L, the clade of green algae diverged into two clusters: a C_3_–C_4_ cluster including both *U. prolifera* and *O. tauri*, which have all the genes involved in the C_4_ pathway, and a C_3_ cluster including *C. reinhardtii* and *V. carteri*. However, PPDK of *O. tauri* was clustered with the genes from land plants, and PPDK of *O. tauri* and *E. vivipara* appears to be more ancient than that of higher land plants. PPDK in *U. prolifera* was clustered with the genes found in the C_3_ green algae (*C. reinhardtii* and *V. carteri*.) and in the C_3_–C_4_ brown alga *T. pseudonana*, and PPDK in *T. pseudonana* appears to be more ancient than that in green algae. Overall, PPDK in green algae also has multiple independent origins as that in land plants.

### Analysis of *rbc*L and PPDK gene expression under various forms of stress

**Figure 3:**
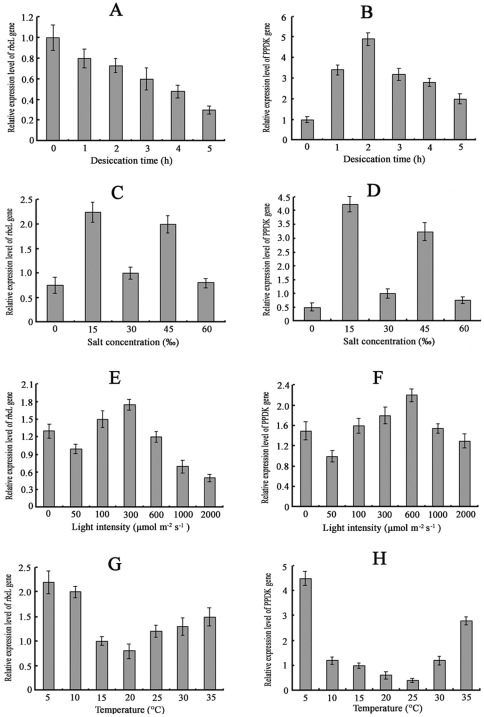
Real-time quantitative PCR analysis for the relative expression level of *rbc*L and PPDK gene in *U. prolifera* subjected to different forms and intensities of stress. Data are means of three independent experiments (±SD). Relative mRNA expression of rbcL and PPDK exposed to different stress conditions: (A, B) desiccation for different durations up to 5 h, (C, D) different salt concentrations for 3 h, (E, F) different light intensities for 3 h, (G, H) different temperatures for 3 h.

Relative quantitative PCR were carried out to determine the differences in expression levels of *rbc*L and PPDK genes under the different stress treatments. Figures 3A and 3B show the profiles of expression of *rbc*L and PPDK as affected by desiccation for varying lengths of time. The expression levels of *rbc*L and PPDK under normal conditions were taken as 1. The expression levels of *rbc*L decreased slowly with time, whereas those of PPDK increased steadily at first, peaking (a 4.9-fold increase) at 2 h, and decreased thereafter. Levels of salinity affected the expression markedly compared to that under normal salinity (30‰), which was taken as 1. The transcript levels of both *rbc*L and PPDK increased at lower and higher levels of salinity but then decreased at very high and very low salinity (Fig. 3C and 3D). Changes in expression levels under different light intensities are shown in Figures 3E and 3F. For each gene, the expression under 50 µmol m^−2^ s^−1^ was taken as 1. The expression level of *rbc*L in the dark was similar to that under normal light intensity, whereas that of PPDK was up-regulated 1.5-fold in the dark. The expression level of *rbc*L peaked at 300 µmol photons m^−2^ s^−1^, while that of PPDK peaked at 600 µmol photons m^−2^ s^−1^. Although the expression of PPDK decreased under high light intensity, it was still higher than it was under normal light intensity. Moreover, the effect of light intensities on PPDK was significantly higher than it was on *rbc*L. The expression of *rbc*L and PPDK at normal temperature (15°C) was taken as 1. The expression levels of *rbc*L reached the lowest point at 20°C, whereas those of PPDK were reached at 25°C. The expression of both rose at both higher and lower temperatures (Fig. 3G and 3H).

### Activity of RuBP carboxylase and PPDK

**Figure 4:**
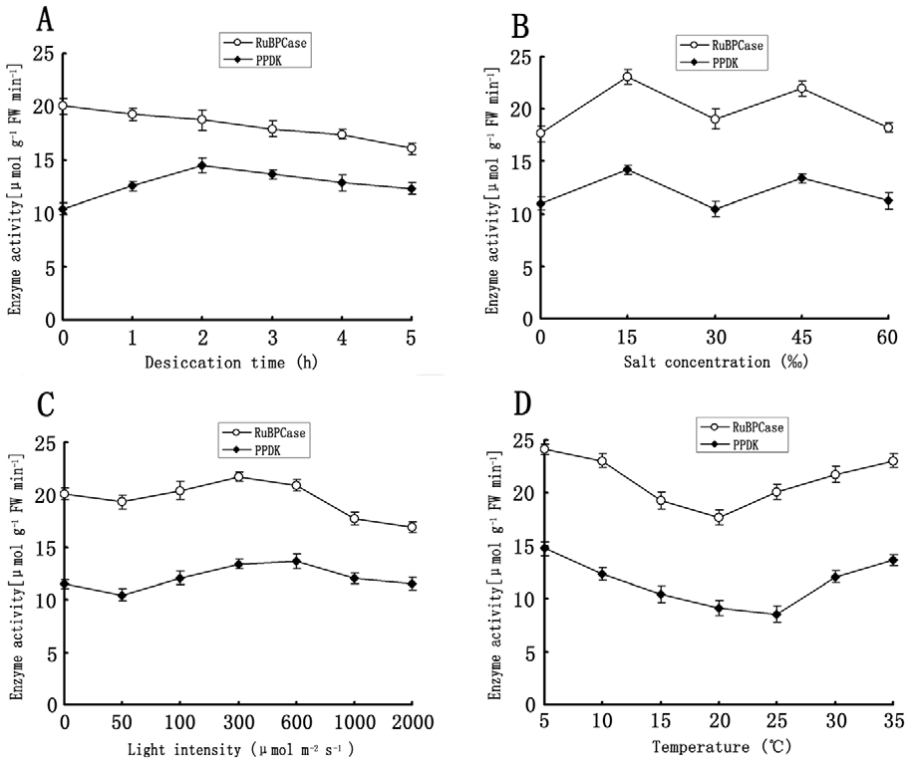
Activity of RuBP carboxylase and PPDK in *U. prolifera* exposed to different forms and intensities of stress: (A) desiccation for different durations up to 5 h, (B) different salt concentrations for 3 h, (C) different light intensities for 3 h, (D) different temperatures for 3 h.

The activity of RuBP carboxylase decreased significantly with the duration of desiccation, whereas that of PPDK increased with the duration up to 2 h, the peak value being 1.4 times the normal value, and decreased thereafter (Fig. 4A). The effects of salinity level on RuBP carboxylase activity and PPDK activity were consistent (Fig. 4B): enzyme activity increased at low and high levels of salinity but then decreased at very low and very high values. Different light intensities clearly influenced the activity of both enzymes in a similar direction: the activity began to rise initially, peaked at 300 or 600 µmol photons m^−2^ s^−1^, and decreased thereafter as light intensity increased further (Fig. 4C). There was almost no difference in the activity of RuBP carboxylase and PPDK between the level under darkness and that under normal light intensity. Temperature also affected both enzymes significantly and similarly (Fig. 4D): RuBP carboxylase reached minimum activity at 20°C and PPDK at 25°C. The activity of both rose with increasing and decreasing temperatures.

### Assay of photosynthetic rate

**Figure 5:**
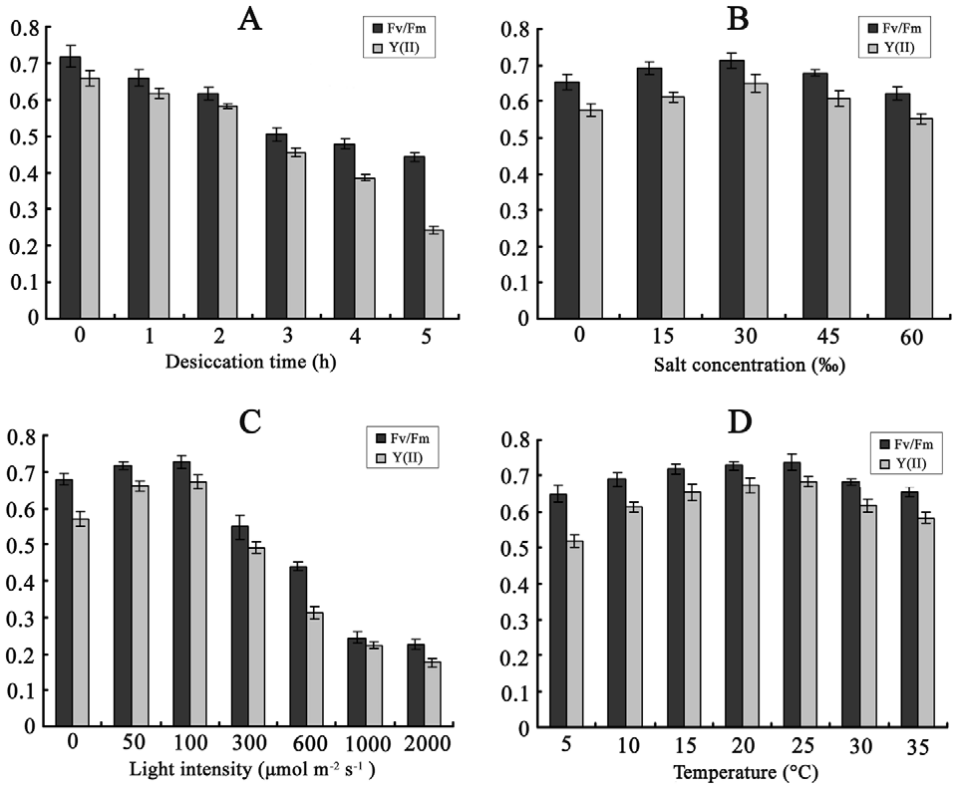
Optimum quantum yield (Fv/Fm) and effective PS II quantum yield (Y II) in *U. prolifera* under different forms and intensities of stress: (A) desiccation for different durations up to 5 h, (B) different salt concentrations for 3 h, (C) different light intensities for 3 h, (D) different temperatures for 3 h.

The optimum quantum yield (Fv/Fm) and effective PSII quantum yield (Y II) reached higher levels under normal conditions (15°C, 50 µmol photons m^−2^ s^−1^) and achieved the maximum values at 25°C, 100 µmol photons m^−2^ s^−1^ (Fig. 5). Neither was markedly affected by salinity or temperature, but both decreased rapidly under prolonged desiccation and high light intensities.

## Discussion

Studies of photosynthetic pathways of marine macroalgae are scanty, and we have a very limited understanding of the mechanisms controlling the altered cell biology and morphology associated with C_4_ *Ulva* species. In the present study, we found that almost all transcripts encoding the proteins required for the core C_4_ cycle have higher steady-state mRNA levels, suggesting that the C_4_ pathway does exist and that the activity of the C_4_ cycle enzymes is controlled at least partially at the level of transcript abundance (Fig. 1). The different expression profiles and product accumulations of *rbc*L and PPDK indicated that these two genes had respectively taken part in C_3_ and C_4_ core cycles under different conditions. We acquired a full-length cDNA sequence of PPDK, a key enzyme of the C_4_ pathway, to gain insights into the evolutionary optimization of C_4_ biochemistry in *Ulva*. The combination of photosynthetic, anatomical, and molecular datasets enabled us to isolate some of the steps in C_4_ evolution and provides fertile new ground for developing hypotheses about anatomical and ecological conditions that promote the evolution of this complex trait.

C_4_ photosynthesis is a series of anatomical and biochemical modifications that concentrate CO_2_ around the carboxylating enzyme Rubisco, thereby increasing photosynthetic efficiency in conditions promoting high rates of photorespiration. C_4_ plants are believed to have evolved gradually from C_3_ plants through several intermediate stages of C_3_–C_4_ plants [52]. However, the evolutionary processes giving rise to C_3_–C_4_ intermediates and C_4_ plants are yet to be elucidated. Phylogenetic analysis of PPDK revealed that C_4_-like photosynthesis in green algae has multiple independent origins (Fig. 2), a result that is consistent with the results from diatoms [19], [53], [54]. Relative studies on diatoms reveal that they have obtained a redundant set of carboxylation and decarboxylation enzymes during complicated endosymbiosis events, which could potentially constitute C_4_-type pathways including lateral-gene transfer (LTG) [54]. Higher plants were exposed to much higher pCO_2_ at the beginning of evolutional history but then became starved for CO_2_ by a steep decrease of CO_2_ and increase of O_2_. These changes were a major driving force for land plants to develop C_4_ metabolism for suppression of photorespiration. Analogous evolutionary events might have taken place in the marine environment without loss of biophysical CCM [55].

Information about C_4_-related enzyme variations under various treatments is considerable. In *Egeria densa*, transfer from low temperature and light to high temperature and light conditions induced increases in the activities and amounts of both PEPC and NADP-ME. After 3 d of treatment, PEPC specific activity increased about 1.7 times relative to values in plants at LTL, whereas NADP-ME activity increased 1.26 times [56]. The submersed monocot *Hydrilla verticillata* is a facultative C_4_ NADP-malic enzyme (NADP-ME) plant in which the C_4_ and C_3_ cycles co-exist in the same cell. The transcript expression of PEPC in *H. verticillata* was substantially up-regulated during light stress [57]. In *U. prolifera*, both C_3_ and C_4_ pathway enzymes exist under normal conditions (Fig. 4). The expression levels of *rbc*L and PPDK increased under stress conditions, such as high salinity, low salinity, high temperature, and low temperature, but the levels of PPDK were higher than those of *rbc*L by 3.25, 4.25, 2.8 and 4.5 times, respectively (Fig. 3). The expression levels of *rbc*L decreased slowly with desiccation time, whereas those of PPDK increased steadily at first and decreased thereafter. These results indicate that both C_3_ and C_4_ cycles may function under normal conditions in *U. prolifera*, while C_4_ photosynthesis may play a more significant role under stress conditions.

**Figure 6:**
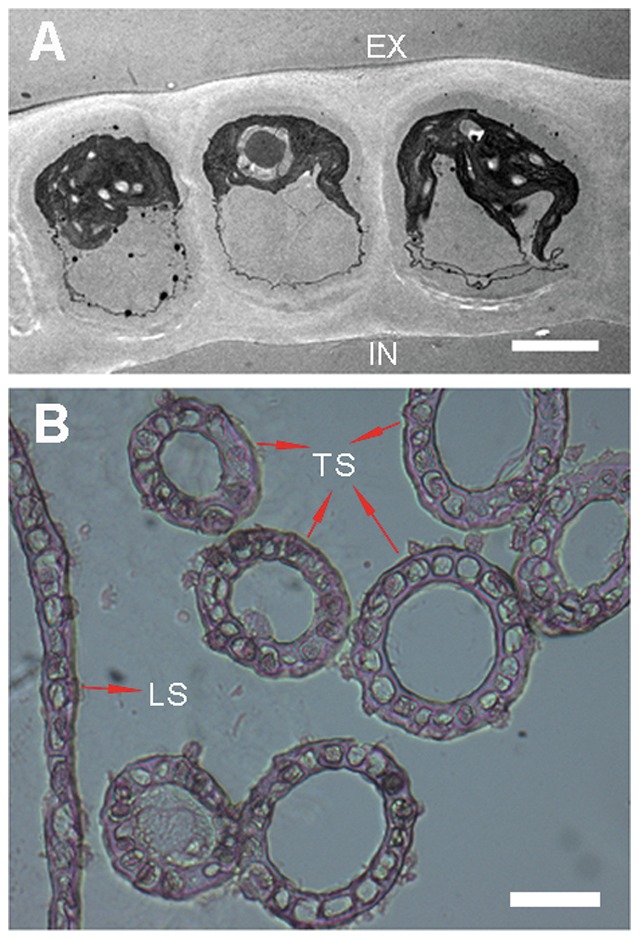
Longitudinal and transverse section view of *U. prolifera*. A, Transmission electron microscopy of transverse section. EX, external of cavity; IN, inner of cavity. Bar, 5 µm. B, Longitudinal and transverse section view with an optical microscope. TS, transverse section; LS, longitudinal section. Bar, 20 µm.

*Ulva prolifera* is a green macroalga with single-layered tubular thalli (Fig. 6A). It differs from most other multi-cellular C_4_ land plants, in which, with few exceptions [58]–[62], the assimilation of CO_2_ is distributed over two cell types, the mesophyll cells (MCs) and the bundle sheath cells (BSCs) [63]. The distribution of CO_2_ assimilation over two distinct cell types requires a massive flux of metabolites between MCs and BSCs [2], [64]. *Bienertia sinuspersici*, a land plant, is a recently discovered species with a unique form of C_4_ photosynthesis. In this single-cell C_4_ species (SCC_4_), the carbon concentrating mechanism does not depend on cooperation between M and BS cells, as it does in Kranz-type C_4_ species. Rather, it possesses a unique chlorenchyma with two functional and biochemically different chloroplast types within photosynthetic cells. Peripheral chloroplasts are spatially separated by a large vacuole from chloroplasts clustered in a central compartment (C-CP). This structural arrangement allows for enrichment of CO_2_ in the Rubisco-containing C-CP, ultimately repressing photorespiration, similar to the mechanism in Kranz-type C_4_ plants. In *U. prolifera*, chloroplasts aggregate lucipetally along the outer side of the layer, and there are apparently no functionally or biochemically different chloroplast types (Fig. 6B), so the chloroplast differentiation mechanism is not fit for this species. Indeed, information about the mechanisms controlling the altered cell biology and morphology associated with C_4_ photosynthesis is very limited. The C_4_ cycle likely affects not only the relatively small number of enzymes and transport proteins needed to perform the core reactions but, given the consequences to the ecological performance of the plants, also a range of other processes [65].

In the present study, the results showed that the expression of PPDK in *U. prolifera* was higher under some daily-encountered stress conditions, such as desiccation, high light intensity, high temperature, and low temperature (Figs. 3, 4). High temperature is a major environmental requirement for C_4_ evolution because it directly stimulates photorespiration and dark respiration in C_3_ plants [66], [67]. The availability of CO_2_ as a substrate also declines at elevated temperature because of the reduced solubility of CO_2_ relative to O_2_ [68]. Aridity and salinity are important because they promote stomatal closure and thus reduce intercellular CO_2_ levels, again stimulating photorespiration and aggravating a CO_2_ substrate deficiency [3]. C_4_ photosynthesis has been found in some marine algae. The implications of marine C_4_ photosynthesis are very significant. The presence of the C_4_ pathway is likely to influence algal sensitivity to changes in CO_2_ concentrations. As in terrestrial ecosystems, C_4_ photosynthesis may therefore be a factor that is shaping species distribution and succession if it occurs in only some members of the phytoplankton. It could operate both on geological timescales and in response to the present rise in atmospheric CO_2_ concentrations. If C_4_ photosynthesis can account for a significant portion of marine carbon fixation in some species, it will affect various aspects of marine ecology and biogeochemistry [69]. C_4_ photosynthesis is a complex biological trait that enables plants to either accumulate biomass at a much faster rate or live in adverse environments compared with “ordinary” plants [40], [70]. Our results suggest that photosynthetic organisms may have evolved a unique mechanism for coping with environmental transition, before losing CCM, and the C_4_ pathway may have first formed in intertidal pluricellular green algae before plants colonized terrestrial habitats. An added benefit of the C_4_ syndrome is improved nitrogen- and water-use efficiencies that have likely contributed to their global distribution and high rates of productivity [71]–[73]. Therefore, the manmade environmental changes, such as CO_2_ rise and eutrophication, stimulate the expression of the C_4_ pathway, while the cooperation of CCM and the C_4_ pathway may enhance the capacity of photosynthesis, which may be one of the most important factors leading to the rapid accumulation of the vast biomass of *U. prolifera* in the green tide that has occurred in the Yellow Sea in four consecutive years since 2008 [27], [31].
